# Utilising quantitative methods to study the intersectionality of multiple social disadvantages in women with common mental disorders: a systematic review

**DOI:** 10.1186/s12939-023-02061-8

**Published:** 2023-12-18

**Authors:** Nadia Alhamd Alghamdi, Kirsty Dunn, Deborah Cairns, Craig Melville

**Affiliations:** 1 Institute of Health and Wellbeing, University of Glasgow, Glasgow, UK; 2 School of Health and Wellbeing, College of Medical Veterinary and Life Sciences, University of Glasgow, Glasgow, UK

**Keywords:** Mental health disorder, Gender/sex, Intersectionality, Social disadvantage

## Abstract

Women are at greater risk of common mental disorders. The intersectionality concept provides a framework to examine the effects of multiple social disadvantages on women’s mental health. We conducted a systematic review to collect and analyse information to identify the quantitative methodologies and study designs used in intersectional research to examine women’s mental health and multiple social disadvantages. Included studies used accepted statistical methods to explore the intersectional effects of gender and one or more types of social disadvantage from the PROGRESS-Plus inequity framework: a place of residence, race/ethnicity, occupation, gender/ sex, religion, education, socioeconomic status, social capital (O’Neill et al. J Clin Epidemiol 67:56–64, 2014). The scope of this systematic review was limited to studies that analysed common mental disorders in women and men comparatively. Studies focusing on only one gender were excluded, ensuring a comprehensive comparative analysis of the intersection of social disadvantages in mental health.

Twelve papers were included in the narrative synthesis (Table 1). Eight of the included papers (67%) reported an intersectional effect of gender and one or more additional types of social disadvantage. The multiplicative effect of gender and socioeconomic status on the risk of common mental disorders was the most commonly reported interaction. This systematic review shows that multiplicative and simultaneous interactions of multiple social disadvantage increase the risk of common mental disorders experienced by women. Moreover, it underlines the potential for quantitative research methods to complement qualitative intersectionality research on gender and mental health. The findings of this systematic review highlight the importance of multiple social disadvantage in understanding the increased risk of mental health experienced by women.

**Supplementary Information:**

The online version contains supplementary material available at 10.1186/s12939-023-02061-8.

## Introduction

Encompassing a state of complete physical, mental, and social well-being [1], women’s health plays an essential role in the health and well-being of modern society. Evidence from existing literature on women’s health suggests that healthier women lead to better-educated and more productive societies, as well as influencing the pace of economic growth and societal development [2]. Further evidence suggests that women’s health also crucially impacts the health and economic well-being of future generations [2]. While it is evident that healthy women are a cornerstone of healthy societies, globally, women continue to experience poor health [3].

A compelling body of evidence has identified social inequalities to have a crucial impact on women’s health and access to health care. These inequalities are based on gender, age, income, race, disability, sexuality, ethnicity, and class, which are considered to shape a woman’s exposure to health risks and access to health services [4–7] & World Health Organization [8]. It is important to acknowledge that these social inequalities rarely impact women’s health in a unilateral way; instead, they experience multiple social disadvantages that substantially impact women’s health.

Health inequality, a global issue, often follows a social gradient where advantaged groups with more resources tend to be healthier [9]. These inequalities extend to mental health, affecting aspects like socioeconomic status, gender, and gender identity [9]. Mental disorders are prevalent in women worldwide [8], with women experiencing a higher rate of internalising disorders such as depression and anxiety than men [10]. Studies show significant differences in mental health across countries and genders, with worse mental health observed in women, lower socioeconomic groups, and individuals with weak support networks [11]. Sexuality and ethnicity also impact mental health [12]. However, the effects of these social determinants on health are intricate and interconnected, extending beyond the simple sum of their individual effects.

The theory of intersectionality is a framework which seeks to understand the interactions between multiple social identities such as race, gender/sex, and class to create unique forms of discrimination, oppression, inequality, and social justice. Originally a Black feminist theory, this approach was the first to propose there was mutuality between factors that could not be understood within the context of research which sought to analyse one factor at a time [13, 14]. With regard to population health, the concept of intersectionality has much to offer. Indeed, it was recently recognised as an important framework for public health in providing accuracy in identifying inequalities, developing intervention strategies, and ensuring results of interventions are relevant within specific communities, outcomes that cannot always be sought from methods focusing on the unitary cause [13].

In qualitative designs within feminist studies, intersectionality provides a vital lens for understanding social phenomena by underlining the interconnected social identities and their affiliated forms of oppression [15]. From an epistemic perspective, intersectionality provides a more thriving, multifaceted analysis that allows for a subtle understanding of lived experiences, giving a voice to individuals who represent intersecting identities [16]. Furthermore, it highlights contextual factors such as socio-political systems and cultural norms that influence these experiences [16]. It allows the researcher to examine and address the layers of inequalities individuals face at the intersections of multiple marginalisation’s [14].

Despite such concerns about people’s lived reality and personal experience, adopting a quantitative approach to investigate intersectionality has several advantages such as estimating the effect measure of statistical relationship between factors or variables. However, employing this theoretically rich technique in population health research nevertheless has limitations and methodological challenges [17]. There are concerns, for example, about how quantitative terms used mathematically in a theoretical study can provoke difficulties concerning how to interpret intersectionality effects in research and how quantitative interpretation will reflect on the analysis and measurement of the outcome results [13]. As such, further investigation is required to determine the most appropriate quantitative approach (es), analytical method(s), and technique(s) for quantitative studies of intersectionality and to determine what would enable the integration of intersectional theory in a way that addresses the inequity about incorporating intersectionality in quantitative research [18].

This systematic review thus collects and examines evidence to do the following:

1. Identify the quantitative methods and study designs used in intersectional research to understand women’s mental health and social disadvantage.
2. Determine whether these techniques help understand women’s mental health and social disadvantage.
3. Analyse and underline the most significant statistical challenges in quantitative intersectionality studies, highlighting methodological limitations and the strength of using the quantitative methods.
4. Assess and classify the overall strength and validity of using an intersectionality framework in quantitative research, focusing on its contributions to women’s mental health and social disadvantage.

## Methods

The systematic review was conducted in adherence with the Preferred Reporting Items for Systematic Reviews and Meta-analysis (PRISMA) guidelines [19] and the Cochrane Handbook for Systematic Review of Interventions [20]. No protocol for this review has been published.

### Search strategy

A search strategy (see Additional file 1: Appendix 1) was developed with the help of a research librarian specialist, and the following electronic databases were searched during December 2019 and updated on 30 June 2022: Medline, EMBASE, CINAHL, PsycINFO, and Sociological Abstract. Hand searching for journals was also conducted during the same period, including articles and papers that suit the search terms in printed journals or general web searches to find niche journals that might not cover academic electronic databases such as *Journal of Mental Health*, *Journal of Health and Social Behaviour*, *Social Science and Medicine* and the *British Journal of Psychiatry*. The search strategy was developed using detailed search terms to capture the essence and key aspects of this systematic review. These aspects include:

1. Gender and its synonyms;
2. Social disadvantage and its synonyms;
3. Intersectionality and its synonyms;
4. Common mental disorders.

Gender keywords were adopted from the ‘Development of a PubMed-Based Search Tool for Identifying Gender and Gender-Specific Health Literature’ [21]. Keywords for ‘common mental disorder’ (CMD) were chosen from the study’ Recruitment and Retention Strategies in Mental Health Trials–A Systematic Review’ [22]. Social disadvantage keywords were developed from the PROGRESS-Plus inequity framework [23]. In collaboration with my primary academic advisor, we carefully selected keywords to cover the meaning of intersectionality. After this preparation, we searched each eligible paper using specific terms such as ‘intersectionality’, ‘intersectional’ and ‘intersection’ along with terms such as ‘inequal’, ‘social inequal’, ‘marginalise’, ‘multiple inequality’, and ‘inequity’.

It is important in this systematic review to broadly define the concepts of sex and gender when examining potential differences between men and women. Sex refers to the biological characteristics that distinguish males, females and intersex, and these characteristics include hormone variations, reproductive organs, and chromosomes [24, 25]. Gender, however, is a psychosocial construct that encompasses societal expectations, roles, relationships, behaviours, attributes, and opportunities considered suitable for men and women [24, 25]. Furthermore, gender identity refers to an individual’s understanding and psychological connection to the societal categories and expectations relating to gender, regardless of whether they accept or reject these expectations [26].

### Study selection

The inclusion and exclusion criteria to determine eligible studies are listed below.

#### Inclusion criteria:

1. *Quantitative Methodologies*: Studies employed one of quantitative method: cross-sectional, observational, cohort, and controlled studies. The main focus of the systematic review is to capture the utilisation of intersectionality in quantitative research techniques. Therefore, only quantitative studies will be considered for incorporation into the analysis.
2. *Participants*: Studies including both male and female participants were considered eligible. This criterion allows exploring gender-based disparities in mental health outcomes across diverse populations.
3. *Age*: Studies has participants aged 18 years and older were included to ensure relevance to adult populations.
4. *Social Disadvantage*: Eligible studies examined the relationship between two or more types of social disadvantage, as defined within the PROGRESS-Plus framework (Place of residence, Race/ethnicity, Occupation, Gender/sex, Religion, Education, Socioeconomic status, Social capital).
5. *Analytical Methodologies*: Studies that used one of the following analytical strategies used in quantitative intersectionality research [17], including studies that do not explicitly adopt the intersectionality framework but still examine the influence of multiple social identities and the intersection effect:
  Statistical interactions
  Moderators in meta-analysis
  Multilevel modelling
  Moderated mediation
  Person-centred methods
  Decomposition analysis.

6. *Outcome*: The study outcomes focused on common mental disorders as defined by the Mental Health Foundation, including Depression, Generalised Anxiety Disorder (GAD), Social Anxiety Disorder, Panic Disorder, Obsessive-Compulsive Disorder (OCD), Post-Traumatic Stress Disorder (PTSD), and Phobias.
7. *Language*: Only studies published in English were included in this review.

#### Exclusion criteria

1. *Qualitative Studies*: Qualitative research was excluded from this systematic review as our focus was on quantitative methodologies.
2. *Conference Abstracts*: Conference abstracts were excluded to ensure that the included studies provided sufficient data for a comprehensive analysis.
3. *Lab-based Studies*: Laboratory-based studies were excluded due to concerns related to lack of these type of studies on capturing intersectionality and social disadvantages.
4. *Single-gender Studies*: Include only males or females are excluded to ensure a comprehensive investigation of social identities and experiences to align with the intersectionality framework. However, it is important to recognise that this decision has limited the number of eligible studies and potentially influenced the results.

Myself (NA) the first author, and the primary supervisor (CM) independently reviewed the titles and abstracts of the identified records. A consensus discussion resolved disagreements about inclusion/exclusion. In the second stage, my supervisor and I independently completed inclusion and exclusion checklists for each full-text paper. The level of agreement was 40%, with a Cohen’s kappa of 0.4, and disagreement was resolved through a consensus discussion.

### Data extraction

A data extraction form was designed specifically to identify certain information (i.e., country, sampling period, sample size, age, gender, participant characteristics, type of social disadvantage) using PROGRESS-Plus (a data extraction file was piloted and revised before final use). This review was designed to examine ways of incorporating intersectionality into quantitative methods regarding social disadvantage and mental health. The first author (NA) extracted data from all included studies, with assistive collaboration from the primary supervisor (CM) who also independently extracted data from half of the included articles. A dual review (NA)/(CM) compared extracted data, and disagreements were resolved through consensus discussion.

### Quality assessment

The quality of the included articles was assessed with the Standard Quality Assessment Criteria – the most suitable tool for evaluating quantitative and qualitative research papers from various fields [27]. This assessment includes 14 questions to evaluate the study (e.g., Is the study design evident and appropriate? Is the objective of the study sufficiently described?). Each study has a summary score in the range of 0-1.0, with a higher score representing better quality.

## Results

**Fig. 1.**
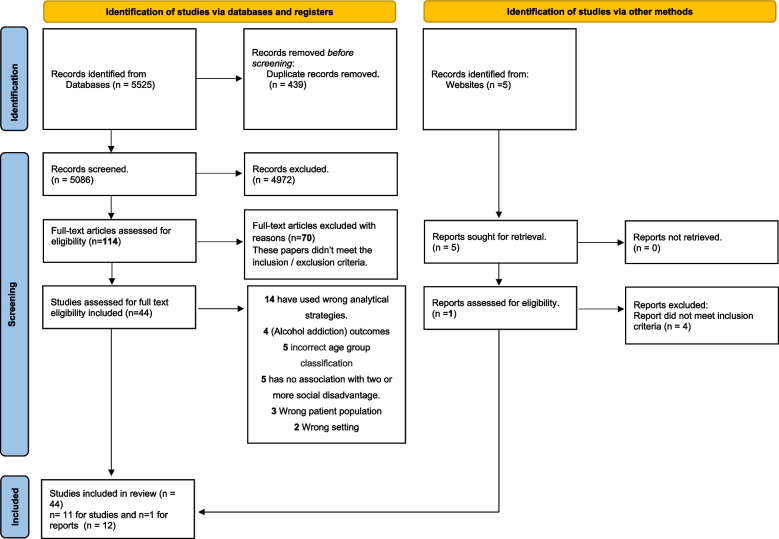
PRISMA flow diagram of the study selection process (2022)

The search strategy generated a total of 5530 potentially relevant articles. Of these, 439 duplicates were identified and removed. Of the remaining 5091 potentially relevant articles, 4972 studies were screened against title and abstract. The resultant 114 papers were assessed for title and abstract review eligibility, and 70 were excluded for not meeting the specified inclusion and exclusion criteria. In sum, 44 articles were considered for full-text eligibility, and 12 were deemed suitable for the narrative synthesis (see the flow diagram in Fig. 1).

### Summary of study characteristics

Of the suitable 12 studies, three were conducted in the United States of America [28–30], two in Scotland [31, 32], one multinational study was conducted in Russia, Poland and Czech Republic [33] and one study was conducted in each of the following countries: Brazil [34], Iran [35], Canada [36], Sweden [37], United Kingdom [38], and Czech Republic [39].

The population’s race was reported in four studies [28, 30, 34, 38], with the reported groups being White, Black, Dark, African American, Native American, Asian, Hispanic and other race/ethnicities. Education level was reported in six studies, with this being school level (primary – university) in four studies [29, 33, 36, 39]. In exploring education, different parameters were used. A single study investigated participant demographics using years of schooling (ranging from less than 4 to more than 12 years) [28]. In two separate studies, separate types were selected, including specified classifications as Low, Medium, or High [30, 34]. Moreover, qualifications were explored, ranging from No qualification to A-level, as employed by Lewis et al. [38].

Employment status was reported in two studies – by manual or non-manual occupation in one study [31] and full-time or part-time in the other [38]. Marital status was reported in seven studies – as single / married /divorced / widowed / never married in three studies [28, 33, 35]; married / cohabitating / formerly married (separated, divorced, or widowed) / never married in two studies [34, 36], couple or single in one study [29], and one-person family units / couples with children / couples without children / single-parent households / respondents living with parents in one study [38]. Financial status was reported in six studies – as low income / not low income in one study [36], report of average net wealth in one study [28], report of financial difficulties and ownership of defined household items in one study [33], household per capita income in one study [34], mean household income in one study [29], and housing tenure and car access in one study [38].

### Type of social disadvantage

Of the twelve overall papers, all reported on gender / gender identity, nine reported on socioeconomic status. Furthermore, six reported on education level, four reported on race / ethnicity, two reported on social cohesion / social ties, one reported on occupation, three reported on age, three reported on social class, and one study reported on marital status (Table 1), farther explanation of each study social disadvantages will be appeared in the summary.

**Table 1:** Characteristics of included studies

Author(s) Year	Title of paper	Country	Study design	Type of data-analytic techniques and methods	Sample size (n)	Gender (Female (%)/ Male (%)/ Other (%)	Type of social disadvantage	Type of CMD	Evidence of intersectionality relevant to gender reported (Yes/ No)
[31]	Ageing, social class and common mental disorders: longitudinal evidence from three cohorts in the West of Scotland	Scotland	Cohort study	Statistical interactions (Hierarchical repeated-measures models)	4510 respondents	Male and female, but the distribution is not specified	• Age • socio-economic status	Anxiety depression	Yes Gender and social class
[39]	Educational inequalities in mental disorders in the Czech Republic: data from CZEch Mental health Study (CZEMS)	Czech Republic	Cross-sectional study	Statistical interactions	3175 respondents	Male (46%) and female (54%)	• Educational attainment • Gender • Age group • Socio-economic status • Health status	Affective disorders Anxiety disorders Alcohol use disorders Substance use disorders	No (There was only an interaction effect between gender and education for substance misuse, but this is not included in the list of common mental disorders in this study)
[35]	Decomposing socioeconomic inequality in poor mental health among iranian adult population: results from the PERSIAN Cohort study	Iran	Cohort study	Statistical interactions	131,813 participants	Male (45%) and female (55%)	• Socioeconomic status • Age group • Physical activity • Gender • Region of residence	Poor mental health	Yes Gender and SES Gender and age Gender and place of residence
[30]	Triple jeopardy? Mental health at the intersection of gender, race, and class	USA	Cross-sectional study	Statistical interactions	7185 respondents	Male (50%) and female (50%)	• Gender • Race • Education	Depression Anti-social problems	No
[36]	Exploring the social determinants of mental health service use using intersectionality theory and CART analysis	Canada	Cohort study	Statistical interactions	1213	Male and female, but the distribution is not specified	• Place of residence • Gender • Education • Socioeconomic status	Depression Social anxiety Panic disorder Phobias Bipolar disorder Substance abuse Agoraphobia	Yes Gender and income
[32]	Social class and gender patterning of Insomnia symptoms and psychiatric distress: a 20-year prospective cohort study	Scotland	Cohort study	Statistical interactions	999	Female: (54.3%) Male: (45.7%)	• Occupation • Gender • Socioeconomic status	Insomnia and Psychiatric distress were determined using the twelve-item General Health Questionnaire	No
[37]	Meddling with middle modalities: a decomposition approach to mental health inequalities between intersectional gender and middle economic groups in northern Sweden	Sweden	Cross-sectional studies	Mediation decomposition analysis	25585	Female: (46%) Male: (54%)	• Gender • Education • Socioeconomic status	Mental health symptoms were assessed using the General Health Questionnaire -12 (GHQ-12)	Yes Gender and income
[28]	Social Ties and depression: An intersectional examination of black and white community-dwelling older adults	United States of America	Cross-sectional studies	Statistical interactions	10441	Female: (59%) Male: (41%)	• Race • Ethnicity • Culture • Gender • Socioeconomic status	Depression	Yes Gender and race
[34]	Social inequalities in the prevalence of common mental disorders in adults: a population-based Study in Southern Brazil	Brazil	Cross-sectional studies	Statistical interactions	1720	Female (55.5%) Male (44.5%)	• Race • Gender • Education • Socioeconomic status	CMD: common mental disorder	No
[33]	Socio-economic status over the life course & depressive symptoms in men & women in eastern Europe	Russia, Poland & Czech Republic	Cohort study	Statistical interactions	25635	Female: (47%) Male:(forty-two%)	• Place of residence • Gender • Education • Socioeconomic status	CMD: common mental disorder	Yes Gender and SES
[29]	Income inequality among American states and the incidence of major depression	United States of America	Longitudinal study	Multilevel modelling	34653	Female: (52.1%) Male (47.9%)	• Place of residence • Race • Ethnicity • Gender • Social capital	Depression	Yes
[38]	Socioeconomic status, standard of living, and neurotic disorder	United Kingdom	Cross-sectional studies	Statistical interactions	9570	Female (54%) Male: (47%)	• Occupation • Gender • Education • Socioeconomic status • Social capital	CMD: common mental disorder	Yes Gender and Social Class

### Type of common mental disorders

All the studies reported affective disorders as common mental disorders, including depression / depressive symptoms / depressive mood / depressive thoughts, reported by [28–31, 33, 34, 36, 37, 39], dysthymia [30, 39], hypomanic and manic episodes [39] as common mental disorders. Studies also reported anxiety disorders, including social anxiety disorder by [33, 34, 36, 39], anxiety/general anxiety disorder [31, 39], panic disorder [36, 39], obsessive-compulsive disorder [39], agoraphobia [36, 39].

Multiple studies reported personality/behaviour disorders such as neurotic psychiatric disorder [38], anti-social personality disorder [30], conduct disorder [30], and bipolar disorder [36]. Furthermore, two studies [36, 39] reported substance use disorders,1 study reported insomnia [32],one study reported somatic symptoms and decreased in vital energy [34], 1 study reported anxiety and alcohol use disorders [39], one study reported phobias [36], one study reported psychiatric distress [32], and 1 study [35] reported poor mental health as a general description for common mental disorders.

### Summary of study design

Twelve papers were accepted for inclusion within the narrative synthesis, of which five studies were cross-sectional studies [28, 34, 37–39], five studies were cohort studies [31–33, 35, 36], one study was a longitudinal study [29], and one study was a secondary analysis [30].

### Summary of type of data-analytic techniques and statistical methodology

Ten studies used statistical interactions [28, 30–36, 38, 39], one study used mediation decomposition analysis [37], one study used multilevel modelling [29].

### Summary of the social disadvantage and mental health effects in the studies

#### Socioeconomic status

Principally, social inequality involves variables that interact with gender, potentially increasing the likelihood of a mental disorder – particularly among women. In this context, 11 out of the 12 studies examined the aspect of socioeconomic status [28, 29, 31–39]. Among these, seven studies demonstrated an interaction between lower socioeconomic status and the presence of a mental disorder, a relationship that becomes more pronounced when women originate from middle or lower-income backgrounds [29, 31, 33, 35–38].

Gustafsson et al. [37] found that mid-income women reported poorer mental health than mid-income men and high-income women. Certain studies have also found statistical interactions with more specific mental disorders. Nicholson et al. [33] discovered a significant correlation between social inequality and depression in Eastern Europe, linked explicitly to economic circumstances. Their findings showed that women consistently conveyed higher levels of depression than men across all levels of the trajectory variable. This pattern held valid for every country within Eastern Europe. Furthermore, women who experience psychiatric distress and are socioeconomically disadvantaged were additionally more likely to experience chronic insomnia symptoms, with psychiatric distress more likely to recur or still be present 20 years later than that for higher socioeconomic groups. Applying quantitative methods to socioeconomic status, standard of living and neurotic disorders in the context of the United Kingdom, Lewis et al. [38] found a strong univariate relation between several socioeconomic variables and the prevalence of neurotic disorders in women, and this has also been reflected in usage of clinical facilities. For example, Cairney et al. [36] found that women were significantly more likely to have pursued any clinical service with respect to mental health (OR 1.4, *p* < 0.001), and the authors’ predictive CART analysis modelling indicated that low-income women aged 23–46 were most likely to use mental health services. Also, Najafi et al. [35] found that people with lower socioeconomic status were more likely to have poor mental health, as there was a slightly higher concentration of poor mental health among less-advantaged participants. In a Scottish cohort, Green et al. [32] identified an association between gender and psychiatric distress, with women in late middle age more likely to develop insomnia symptoms than men. Furthermore, Pabayo et al. [29] reported a significant cross-level interaction between depression and lower-income women.

#### Race/ethnicity

The addition of race / ethnicity as an interaction in the aetiology of mental disorders has been identified in the current review. Four studies demonstrated an interaction between race / ethnicity and mental disorder, especially when present in women with lower socioeconomic status [28–30, 34]. With a Brazilian cohort, Moraes et al. [34] identified that common mental disorders are more prevalent in women at lower levels of income, at lower levels of education, and who are Black. In the context of ‘triple jeopardy’ as a minority, Mair [28] observed that Black women might face increased vulnerability due to increasing age, which intersects with being an older woman. This demographic intersection could potentially lead to compounded challenges. Furthermore, race/ethnicity has been identified as a significant determinant in mental health disparities. Rosenfield’s [30] study found that Black females in lower social classes experienced more significant mental health disadvantages than white males in higher social positions.

#### Educational level

Findings on the interaction between educational level and common mental disorders were similar to those on socioeconomic status. Five studies [28, 33, 34, 38, 39] reported a high association of no education / low education level with common mental disorders, as opposed to lower rates of common mental disorders in persons with high school and/or university / graduate education.

#### Age/marital status

Regarding age, only one study shows interaction: Najafi et al. [35] found that older adults more likely experience common mental health disorders as opposed to younger persons. Moreover, regarding marital status the systematic findings align with the observations of Najafi et al. [35], who recognised a correlation between poor mental health and marital status among Iranian adults. It is important to note that although the current study’s findings align with the authors results, not all the examined cases offer specific details.

#### Social class

The interaction between social class and common mental disorders was explored by Green and Benzeval [31], who found a non-linear decrease in the prevalence of anxiety with age and a non-linear increase in the prevalence of depression with age in people within the manual social class as opposed to those in the non-manual social class. Lewis et al. [38] found that people of lower social class status – particularly men – were associated with a higher prevalence of neurotic disorders. Lewis et al. [38] also found a strong correlation between social class and the prevalence of neurotic disorders in people aged 40–54 years.

#### Place of residence

Only one study has shown interaction between a place of residence and poor mental health. Najafi et al. [35] discovered that people living in Sistan and Balouchestan provinces in Iran had the largest prevalence of poor mental health. All 12 studies recognised gender/gender identity [28–39]. However, only eight studies showed strong interactions of gender as a variable with common mental disorders. All these studies found that women and girls were more at risk of experiencing and being diagnosed with common mental health disorders than men and boys,irrespective of other factors such as race/ethnicity, education or socioeconomic status, gender is an extremely predominant and strong factor.

### Summary of statistical measurement

In this systematic review, nine of the 12 studies reported statistically significant findings (summarised in this section), offering valuable insights into social disadvantage and mental health intersections. Analysing the social determinants of mental health service using intersectionality theory and a CART analysis [36], the main effects model (logistic regression) revealed that being female was significantly associated with seeking any service for mental health (OR 1.4 *p* < 0.001). Gustafsson et al. [37] found that mid-income women experienced poorer mental health compared with both mid-income men (effect size: 0.42, 95% CI: 0.14–0.71, *p* < 0.001) and high-income women (effect size: 0.44, 95% CI: 0.13–0.74, *p* < 0.001). Also, Mair [28] showed the moderating effect of race and gender on the relationship between social ties and depressive symptoms, with the three-way interaction effects explaining 22.4% of the variation. Moraes et al. [34] identified a higher prevalence of common mental disorders among females, Blacks with lower education and income levels, and those who are divorced, separated, or widowed, with females having a CMD prevalence of 20.5% compared to 7.4% among males. Nicholson et al. [33] consistently found higher odds ratios for depressive symptoms in women than men across all trajectory variables in all Eastern European countries, with odds ratios for depressive symptoms being 2.03 (95% CI: 1.75–2.35) for women and 2.10 (95% CI: 1.82–2.43) for men in Russia, 2.31 (95% CI: 2.03–2.62) for women and 2.39 (95% CI: 2.12–2.71) for men in Poland, and 1.64 (95% CI: 1.40–1.94) for women and 1.79 (95% CI: 1.53–2.08) for men in the Czech Republic. Higher-income inequality was associated with an increased risk of depression among women in the fourth quintile (OR 1.37, 95% CI 1.03 to 1.82) and the fifth quintile (OR 1.50, 95% CI 1.14 to 1.96), and women in states with higher-income inequality had a higher risk of developing depression [29]. Lewis et al. [38] observed a significantly higher prevalence of neurotic disorder in women with no qualifications than those with higher educational attainment (odds ratio: 1.26, 95% CI: 1.06–1.49). Green and Benzeval [31] reported a higher prevalence rate of anxiety among females (34.8%), while depression among females was 12.0% in the West of Scotland. Lastly, Najafi et al. [35] revealed a higher prevalence of poor mental health in women (17.2%) compared with men (13%), indicating a gender difference in the prevalence of mental disorders – that is, to reiterate, a higher rate for women. The reporting of effect measures and statistical results provides a quantitative understanding of variable associations, while the complex interplay among social disadvantage factors and mental health outcomes increases the clarity and comprehensiveness of the systematic review’s findings.

## Discussion

This systematic review has sought to address a crucial gap in quantitative intersectionality by focusing on the methods for incorporating intersectionality into understandings of mental health disorders among women.

### An overview finding on quantitative methods of intersectionality and its effects in women mental health studies

The findings from previous studies have observed consistent associations between mental disorders and social disadvantage in women. However, the results of this systematic review highlight that the cause of mental disorders in women is not simply the effect of an independent unitary social disadvantage. Instead, it emerges from complex multiplicative and simultaneous interactions, emphasising the indispensability of intersectionality approaches in comprehending and addressing the dynamic health issues relating to gender [17].

The term ‘intersectionality’ was initially introduced by Kimberle Crenshaw in 1989 [14], though the first quantitative intersectionality paper appeared 11 years later in 2001. Bauer et al. [40] stated that quantitative intersectionality analyses offer to explore the research data and the statistical effect on both micro and macro aspects of health issues, solving complicated multiplicative and compound effects within large datasets [40]. This quantitative approach to the theory enables policymakers to create strategies for addressing the root causes of health disparities. By illustrating the impact of social disadvantages on mental health disparities in women through statistical techniques involving numbers and percentages, this approach offers a more precise description of intersectionality’s impact.

The concept of intersectionality, implanted in feminist theory and discussed by scholars like Hancock [41], provides a crucial framework for understanding marginalised women [15]. While it is predominantly explored through qualitative research, this systematic review used a quantitative perspective on intersectionality to identify useful findings such as those from the quantitative analysis of Moraes et al. [34], which indicated the complex relationship between lower education and income levels and their impact mental health outcomes. Validating the power of quantitative methodologies offers much to feminist theory in terms of evidencing on the social factors that shape women’s mental health experiences.

This systematic review, as noted, identifies the quantitative methods and study designs used in intersectional research to understand women’s mental health and social disadvantage and assesses whether particular techniques were beneficial for understanding social disadvantage effects on mental health disorders among women. The findings from the study show that the most commonly used statistical methods were statistical interactions, mediation decomposition analysis, and multilevel model. Statistical interactions, a method in intersectional research, focus on how two or more categories interact to create combined effects, these interaction terms allow for examining the joint influences of variables and can be implemented as multiplicative or additive effects to explore how variables interact and shape the outcomes [16] For example, discrimination against Black women is more than just the sum of racism and sexism; in fact, its multiplicative effects imply the descriptions of discrimination when one factor’s impact depends on another factor’s existence [18]. Statistical interactions thus allow research to comprehend the multiplicative effects of multiple variables, such as gender, socioeconomic status, and race, on mental health outcomes [13]. Mediation decomposition analysis allows for the breakdown of the total effect of gender on mental health into direct and indirect effects through socioeconomic status. Because of this approach, the study explored how socioeconomic factors mediate or explain the gender inequality observed in mental health outcomes. Integrating mediation analysis adds depth to understanding how specific social categories contribute to the mental health landscape [37]. Multilevel modelling captures the complex interactions between social disadvantage factors at both individual and contextual levels, and this offers insights into the interpretations of mental health outcomes across different groups [29]. This is consistent with the findings of previous reviews that examined the use of quantitative methods to explore the intersectionality of multiple social disadvantages for various outcomes, both with and without a focus on gender [40, 42].

The strategic use of quantitative techniques not only facilitated the extraction of data from the studies but also enabled a robust analysis of the multiplicative effects of social disadvantages on women’s mental health [40]. Employing quantitative methods to analyse intersectionality thus helps to present accurate statistical data, including prevalence and numerical figures, which are significant indications of effective research; statistical data works as compelling evidence that guides the policymaker in terms of practical solutions, such as implementing specific policies for improving women’s economic status, addressing gender inequalities, and promoting mental health outcomes for marginalised women. An ideal example from the systematic review comes from a study by Lewis et al. [38], which investigated complex socioeconomic indicators on neurotic disorders using statistical interactions. The authors observed a significantly higher prevalence of neurotic disorder in women with no qualifications than those with higher educational attainment (odds ratio: 1.26, 95% CI: 1.06–1.49). The same study also provided other valuable insights: this analysis revealed that 10% of neurotic disorders are linked to living standards. Such insights assist policymakers in designing targeted interventions that address specific socioeconomic inequality and enhance public health to improve mental health outcomes for women as well as men nationwide.

This systematic review facilitates understanding of if and how particular quantitative techniques help identify factors relating to social disadvantage effects on women’s mental health. In the analyses of 12 reviewed studies, nine have shown that the most significant variable contributing to social disadvantages affecting women’s mental health is socioeconomic status [28, 29, 33–39]. The analysis by Moraes et al. [34] found a prevalence of 20.5% of common mental disorders in women with lower education levels and occupations with lower socioeconomic status compared to 7.4% among men. Quantitative data on social inequality (e.g., income level, education, and occupation) is linked to a high prevalence of common mental disorders in women. Results help identify these inequalities’ causes and potential solutions [13]. This finding does not imply homogeneity in the employed methodologies, or the populations studied. The studies included in the systematic review showed heterogeneity in various aspects, such as sample sizes, study designs, measurement tools, and demographic characteristics. The differences in the studies influence how the findings are used in other contexts or populations. Solutions can also be extracted from quantitative results of intersectionality analyses, such as improving marginalised women’s educational opportunities and enhancing their skills as these two endeavours will elevate their financial income and give them independence, which will help improve their mental health.

A synthesis of the systematic review findings reveals a significant and consistent interaction between socioeconomic status, gender, and race/ethnicity that affects common mental disorders. Women from ethnic minorities with lower socioeconomic face compound challenges and experience increased rates of mental disorders. Therefore, incorporating a quantitative intersectional lens will help to investigate the accumulative impact of various social indignities to determine the root causes of mental health disorders among women. Such actions can help policies and programmes tackle gender inequality and effectively promote mental health and well-being among marginalised women.

### Strengths and limitations

The studies included in the systematic search have various methodological strengths and weaknesses that might have influenced the outcomes reported. One of the strengths of the systematic review is the heterogeneity of methodologies and population of the included studies, which is important to consider when interpreting the findings. The review highlights consistent trends and associations between social disadvantage and mental health outcomes, and the diversity in sample sizes and study designs, such as cross-sectional, cohort and longitudinal studies, along with the use of various measurement tools to estimate common mental disorders and social disadvantage variables, and indeed the demographic characteristics, including age, race/ethnicity, and socioeconomic status, all offer much to this review (Table 1). Specifically, for example, certain variations show diversity in the factors that influence mental health and underline the complexity of intersectionality relations between social disadvantage and mental health outcomes.

Furthermore, the restricted number of studies found reflect the limited quantitative research conducted and the challenges posed through the integration of quantitative methods to look at research problems typically addressed by qualitative methods. However, the limited number of studies found could also be because studies that examined heterogeneity from the perspective of social disadvantage exclusively within the group of women were not included.

Nevertheless, the results of the findings do agree with pre-existing qualitative literature and enhance understanding of the area of women’s mental health. Value has been demonstrated in using a quantitative method application to understand intersectionality within this space and should be encouraged to continue with a focus on homogeneity in quantitative approaches to facilitate qualitative and quantitative synthesis ability within the area in future. Both approaches would provide a powerful data set in order to inform clinical approaches to prevention and treatment.

Our results indicate that self-reported data collection methodology is preferred with such study designs, being used within ten of the 12 studies [28, 31–39]. Self-reported data is a common method for gathering information about individuals’ social identities. Self-reported data means that participants in these studies are asked to describe their social identities, such as gender, race, ethnicity, and socioeconomic status. Their interpretations of personal identity influence people’s self-reporting, which can present perceptions bias in reporting the data [43].

In conclusion, to the best of the researcher’s knowledge this systematic review is the first to collectively analyse the quantitative methods and study designs used in quantitative intersectional research on mental health disorders among mixed-gender populations to determine whether these techniques are practical and effective for quantitative intersectionality research. The 12 observational studies included in this review highlight the use of quantitative intersectionality methods to explore the complex relationships among various social disadvantages and effects on common mental disorders. By representing accurate statistical results, quantitative intersectional research can guide policymakers to improve women’s mental health.

In light of the findings from the systematic review, future research is recommended to explore the intersection of social inequalities’ effects on women’s mental health using the quantitative technique of intersectionality with large-scale data. The accurate statistical results can lead to a better understanding of women’s social identity from an intersectionality perspective and help to address social inequalities and thereby improve women’s mental health.

### Supplementary Information

**Additional file 1.** The following MEDLINE search strategy was used and was adapted as appropriate for other databases.

## Data Availability

Not applicable.
