# Supplementary material for: Utilising quantitative methods to study the intersectionality of multiple social disadvantages in women with common mental disorders: a systematic review
Source: Int J Equity Health. 2023 Dec 18;22:264. doi: 10.1186/s12939-023-02061-8 (PMC10729432; doi:10.1186/s12939-023-02061-8)
Supplement: Supplementary file 1 — Additional file 1. The following MEDLINE search strategy was used and was adapted as appropriate for other databases. [file 12939_2023_2061_MOESM1_ESM.pdf]

Search Strategy:

Appendix 1

The following MEDLINE search strategy was used and was adapted as appropriate for other databases.

Search ID	Search Term	Action
1.	sex based.mp.	1037
2.	sex factors.tw.	213
3.	sex distribution.tw.	3615
4.	sex characteristics.tw.	601
5.	sex dimorphism.tw.	363
6.	gender difference$.tw.	24697
7.	female.tw.	532777
8.	gender.tw.	237153
9.	sex.tw.	400957
10.	wom?n.tw.	931378
11.	1 or 2 or 3 or 4 or 5 or 6 or 7 or 8 or 9 or 10	1799667
12.	Intersection$.tw.	10027
13.	Intersectional$.tw.	571
14.	Inequal$.tw.	19908
15.	Social inequal$.tw.	2474
16.	Social disadvantag$.tw.	862
17.	Marginaliz$.tw.	3784
18.	Multiple inequalit$.tw.	7
19.	Inequit$.tw.	6398
20.	12 or 13 or 14 or 15 or 16 or 17 or 18 or 19	39486
21.	mental disease/ or mental patient/	5921
22.	chronic mental illness/ or exp mental disorders/	1141939
23.	((mental$ or psychologic$) adj2 (deficien$ or disease$ or disorder$ or disturbance$ or dysfunction$ or health or illness$ or problem$)).ti,ab,id.	158545

24.	anxiety/ or exp anxiety disorder/	139230
25.	(anxiet$ or anxious$ or ((chronic$ or excessiv$ or intens$ or (long$ adj2 last$) or neuros$ or neurotic$ or ongoing or persist$ or serious$ or sever$ or uncontrol$ or un control$ or unrelent$ or un relent$) adj2 worry)).ti,ab.	147672
26.	(body dysmorphic disorder or compulsions or compulsive behavior or obsessive behavior).sh. or (body dysmorphi$ or clean$ response$ or compulsion$ or dysmorphophobi$ or imagine$ ugl$ or obsession or obsessional or obsessions or obsessive compulsive or obsess$ ruminat$ or ocd or osteochondr$ or recurr$ thought$ or scrupulosity or ((arrang$ or check$ or clean$ or count$ or hoard$ or order$ or repeat$ or symmetr$ or wash$) adj compulsi$)).ti,ab.	31745
27.	Panic.mp. or panic$.ti,ab. [mp=title, abstract, original title, name of substance word, subject heading word, floating sub-heading word, keyword heading word, protocol supplementary concept word, rare disease supplementary concept word, unique identifier, synonyms]	17310
28.	$phobi$.ti,ab.	9777
29.	(critical incident stress or emotional trauma or psychological stress or stress, psychological or traumatic neurosis).sh. or (acute stress or asd or combat neuros$ or combat syndrome or concentration camp syndrome or desnos or ((extreme or psycho$) adj (stress$ or trauma$)) or flash back$ or flashback$ or hypervigilan$ or hypervigilen$ or posttrauma$ or post trauma$ or ptsd or railway spine or (rape adj2 trauma$) or re experienc$ or reexperienc$ or stress disorder$ or torture syndrome or (traumatic adj (neuros$ or stress)) or (trauma$ and (avoidance or birth$ or death$ or emotion$ or grief or horror or nightmare$ or night mare$))).ti,ab.	208646
30.	exp eating disorder/ or exp eating disorders/	28257
31.	(anorexi$ or bing$ or bulimi$ or (compulsive adj2 (eat$ or vomit$)) or (eating adj2 disorder$) or ednos or ((forced or self induc$ or selfinduc$) adj2 (purg$ or vomit$)) or hyperorexia or over eat$ or overeat$ or (restrict$ adj2 eat$)).ti,ab.	51236
32.	exp mood disorder/	112885
33.	depression/ or exp mood disorders/	206810
34.	exp affective disorders/	112885
35.	((affective or mood) adj (disorder$ or disturbance$ or dysfunction$)).ti,ab.	28704

36.	(((bipolar or bi polar) adj5 (disorder$ or depress$)) or ((cyclothymi$ or rapid or ultradian) adj5 cycl$) or hypomani$ or mania$ or manic$ or mixed episode$).ti,ab.	40703
37.	"explode schizophrenia"/ or (psychosis$ or psychotic$).mp. [mp=title, abstract, original title, name of substance word, subject heading word, floating sub-heading word, keyword heading word, protocol supplementary concept word, rare disease supplementary concept word, unique identifier, synonyms]	72067
38.	exp psychotic disorders/ or exp schizophrenia/ or "schizophrenia and disorders with psychotic features"/	135921
39.	exp psychosis/ or exp schizophrenia/	135921
40.	(a?athisi$ or hebephreni$ or (neuroleptic$ and ((malignant and syndrome) or (movement adj2 disorder))) or oligophreni$ or psychotic$ or psychos?s or schizo$ or (tardiv$ and dyskine$)).ti,ab,id. or ((parkinsoni$ or neuroleptic induc$).ti,ab,id. not (parkinson$ and disease).ti.) or (delusion$ or hallucinat$ or paranoi$ or psychiatric$ or thought disorder$).ti,ab.	313890
41.	exp personality disorder/	39400
42.	borderline states/ or exp personality disorders/	39400
43.	(((aggressiv$ or anxious$ or borderline$ or dependent$ or eccentric$ or emotional$ or immature or passiv$ or psychoneurotic or psycho neurotic or unstable) adj5 personalit$) or (anal$ adj (personalit$ or character$ or retentiv$)) or aspd or character disorder$ or (personalit$ adj5 disorder$)).ti,ab.	22668
44.	(anankastic$ or asocial$ or avoidant$ or antisocial$ or anti social$ or compulsiv$ or dissocial$ or histrionic$ or narciss$ or neuropsychopath$ or obsessiv$ or paranoi$ or psychopath$ or sadist$ or schizoid$ or schizotyp$ or sociopath$ or (moral adj2 insanity)).ti,ab.	74423
45.	(cluster a or cluster b or cluster or ((anxious$ or dramatic$ or eccentric$ or emotional$ or fearful$ or odd$) adj5 cluster$)).ti,ab.	124834
46.	exp alcohol abuse/ or exp drug dependence/ or exp drug abuse/ or substance abuse/	261592
47.	drug seeking behavior/ or exp substance-related disorders/	262116
48.	(alcoholi$ or (alcohol$ and (abstinence or detoxification or intoxicat$ or rehabilit$ or withdraw$))).id,hw.	112684
49.	(alcoholi$ or drinker$1 or (drink$ adj2 use$1) or ((alcohol$ or drink$) adj5 (abstinen$ or abstain$ or abus$ or addict$ or attenuat$ or binge$ or crav$ or dependen$ or detox$ or disease$ or disorder$ or excessiv$ or harm$ or	138902

	hazard$ or heavy or high risk or intoxicat$ or misus$ or overdos$ or over dos$ or problem$ or rehab$ or reliance or reliant or relaps$ or withdraw$)) or (control$ adj2 drink$) or sobriet$).ti,ab.	
50.	(cannabis or cocaine or hashish or heroin or marihuana or marijua$ or ((acetomorphine or amphetamine$ or amphetamine$ or analeptic$ or crack or crank or dextroamphetamine$ or diacephine or diacetylmorphine or diacetylmorphine or diamorphin$ or diamorphine or diaphorin or drug or methadone$ or methamphetamine$ or morfin$ or morphacetin or morphin$ or naltrexone or narcotic$ or opioid$ or opium or polydrug$ or psychostimulant$ or speed or stimulant$ or stimulant$ or substance or uppers) adj3 (abstain$ or abstinen$ or abus$ or addict$ or (excessive adj use$) or dependen$ or (inject$ adj2 drug$) or intoxicat$ or misus$ or over dos$ or overdos$ or (use$ adj (disorder$ or illicit)) or withdraw$)) or ((drug or substance) adj use$)).ti,ab,hw,id.	227237
51.	(hysteria or somatoform disorder or somatization).ti,ab,hw. or (briquet or hysteria* or poly?symptom* or multi?somat* or (multiple and (mups or medically unexplained or unexplained symptoms or physical symptoms or symptom diagnos*)) or somatiz* or somatis*).ti,ab.	11100
52.	21 or 22 or 23 or 24 or 25 or 26 or 27 or 28 or 29 or 30 or 31 or 32 or 33 or 34 or 35 or 36 or 37 or 38 or 39 or 40 or 41 or 42 or 43 or 44 or 45 or 46 or 47 or 48 or 49 or 50 or 51	1875125
53.	11 and 20 and 52	2021
54.	limit 53 to (english language and humans and yr="1990 -Current")	1872
